# Genome-wide identification and evolutionary analysis of RLKs involved in the response to aluminium stress in peanut

**DOI:** 10.1186/s12870-021-03031-4

**Published:** 2021-06-21

**Authors:** Xin Wang, Ming-Hua Wu, Dong Xiao, Ruo-Lan Huang, Jie Zhan, Ai-Qin Wang, Long-Fei He

**Affiliations:** 1 National Demonstration Center for Experimental Plant Science Education, College of Agriculture, Guangxi University, Nanning, 530004 China; 2 Guangxi Key Laboratory for Agro-Environment and Agro-Product Safety, Nanning, 530004 China; 3 Key Laboratory of Crop Cultivation and Tillage, GuangxiColleges and Universities, Nanning, 530004 China

**Keywords:** Peanut, *RLK*, Gene family, Genome-wide analysis, Al stress

## Abstract

**Background:**

As an important cash crop, the yield of peanut is influenced by soil acidification and pathogen infection. Receptor-like protein kinases play important roles in plant growth, development and stress responses. However, little is known about the number, location, structure, molecular phylogeny, and expression of RLKs in peanut, and no comprehensive analysis of RLKs in the Al stress response in peanuts have been reported.

**Results:**

A total of 1311 *AhRLK*s were identified from the peanut genome. The AhLRR-RLKs and AhLecRLKs were further divided into 24 and 35 subfamilies, respectively. The *AhRLK*s were randomly distributed across all 20 chromosomes in the peanut. Among these *AhRLK*s, 9.53% and 61.78% originated from tandem duplications and segmental duplications, respectively. The ka/ks ratios of 96.97% (96/99) of tandem duplication gene pairs and 98.78% (646/654) of segmental duplication gene pairs were less than 1. Among the tested tandem duplication clusters, there were 28 gene conversion events. Moreover, all total of 90 Al-responsive AhRLKs were identified by mining transcriptome data, and they were divided into 7 groups. Most of the Al-responsive AhRLKs that clustered together had similar motifs and evolutionarily conserved structures. The gene expression patterns of these genes in different tissues were further analysed, and tissue-specifically expressed genes, including 14 root-specific Al-responsive *AhRLK*s were found. In addition, all 90 Al-responsive *AhRLK*s which were distributed unevenly in the subfamilies of *AhRLK*s, showed different expression patterns between the two peanut varieties (Al-sensitive and Al-tolerant) under Al stress.

**Conclusions:**

In this study, we analysed the RLK gene family in the peanut genome. Segmental duplication events were the main driving force for *AhRLK* evolution, and most *AhRLK*s subject to purifying selection. A total of 90 genes were identified as Al-responsive AhRLKs, and the classification, conserved motifs, structures, tissue expression patterns and predicted functions of Al-responsive *AhRLK*s were further analysed and discussed, revealing their putative roles. This study provides a better understanding of the structures and functions of *AhRLK*s and Al-responsive *AhRLK*s.

**Supplementary Information:**

The online version contains supplementary material available at 10.1186/s12870-021-03031-4.

## Background

Aluminium (Al) is one of the most harmful factors in plant growth in acidic soils, and Al can cause 25% to 80% yield losses depending on the crop [1, 2]. Al signalling induces a series of physiological events in plant cells. The most obvious phenomena of Al toxicity are inhibition of cell elongation in the apical region and induction of programmed cell death (PCD) [3–5]. PCD is an active, orderly, and genetically controlled form of cell death and occurs in plants throughout development and in response to environmental stresses [6]. Early studies found that Al-treatment can enhance Fe^2+^-induced lipid peroxidation and PCD in tobacco cells [7]. For decades, Al-induced PCD has been proven in many plant species including: soybean (*Glycine max*) [8], maize (*Zea mays*) [9], barley (*Hordeum vulgare*) [10], tomato (*Lycopersicon esculentum*) [11]and peanut (*Arachis hypogaea*) [12]. Al-induced PCD is mediated through two cell signal transduction pathways: a mitochondrial-dependent pathway and a nuclear-dominated mitochondrial-independent pathway [5]. However, Al signal information and its transmembrane transduction are unknown. Both pathways use plasma membrane and/or cell wall-localized receptors to sense environmental stimuli and efficiently transduce signals between cells, which perceive and transduce signals to modulate gene expression and/or enzyme activity as well as motility [13]. Receptor-like protein kinase (RLK) play important roles in the process of cell signal transduction, and are involved in a variety of plant physiological processes including: self-incompatibility [14], environmental signal processing [15], organ shape and meristem activity [16], hormone signal transduction [17], PCD [18], and tolerance to oxidative stress [19]. RLKs sense and transduce signals through protein interactions and phosphorylation [20]. Based on the structure of the extracellular domain, RLKs have been classified into several families such as S-RLKs, LRR-RLKs, EGF-RLKs, LecRLKs, TNFR-RLKs and PR5K-RLKs [21].While many RLKs involved in the environmental stress response have been found, few RLKs have been reported to be involved in Al stress response. *WAK1*, which mediates the interaction between the cell wall and cytoplasm and may participate in cell elongation and morphogenesis [22], was the first RLK that was found to be involved in the Al stress response. Theoverexpression of *WAK1* was reported to enhance Al tolerance in Arabidopsis [23]. The results showed that *RLK*s play an important role in Al-induced PCD, but the mechanism of *RLK*s in the regulation of Al-induced PCD is unknown.

Peanuts are an important oil crop worldwide. Al-dependent inhibition of growth causes a reduction in peanut yield in acidic soil. There is no comprehensive analysis of the RLK gene family in the peanut. In the present study, recently released peanut whole genome sequence data (http://peanutgr.fafu.edu.cn/index.php) were utilized to analyse the RLK gene family in peanut. A total of 1311 *AhRLK*s have been identified. The LRR-RLKs and LecRLKs were further divided into 24 and 35 subfamilies, respectively based on a phylogenetic analysis. The evolution and collinearity of *AhRLK*s were investigated. The evolutionary patterns of the RLK gene family were tested by investigating gene duplication events in the peanut. In addition, 90 *AhRLK*s in response to Al stress were identified by transcriptomic analysis, and the expression profiles of *AhRLK*s at different Al treatment time-points were comprehensively determined. These results will provide a basis for further research on the evolution and physiological functions of *AhRLK*s in response to Al stress in the peanut.

## Results

### Identification of AhRLKs in the peanut

To identify the members of AhRLKs in the peanut, we downloaded publicly available peanut genome sequence data and used the Arabidopsis RLK sequence as a query to perform a genome-wide similarity search. After filtration of the sequence, a total of 1311 AhRLKs that contained at least one kinase domain were initially identified, including 548 LRR-RLKs, 274 LecRLKs, 83 cysteine-rich RLKs, 76 EGF RLKs, 49 proline-rich RLKs, 46 s-domain RLKs, 22 TMK-RLKs, 2 TNFR-RLKs, 1 RRO-RICH RLK, 28 RLCK-RLKs, 24 LysM-RLKs, and 158 no obvious domains (Additional files 1 and 2). LRR-RLKs and LecRLKs were considered for further analyses.

### Phylogenetic analysis of LRR-RLKs and LecRLKs in the peanut

**Fig. 1:**
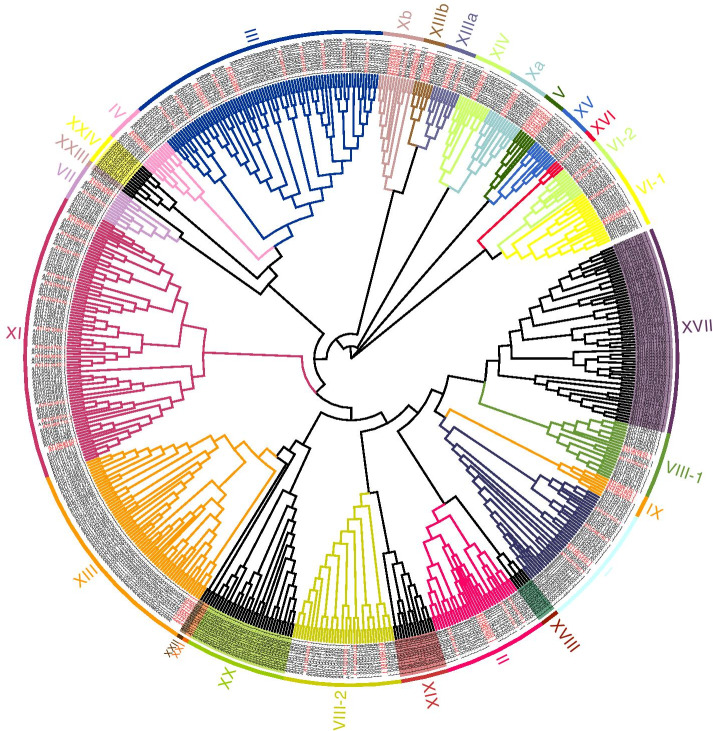
Phylogenetic analysis and classification of peanut and *A. thaliana* LRR-RLK proteins. The phylogenetic tree was established with full sequences using the maximum-likelihood method with 1000 bootstrap replications and the evolutionary distances were computed using the p-distance method. Red sequences indicate the AtLRR-RLKs. Each RLK clade is depicted by a different colour, representing the 24 clades that were identified. Labelled lines on the outside of the tree represent clade names as defined in the text

**Fig. 2:**
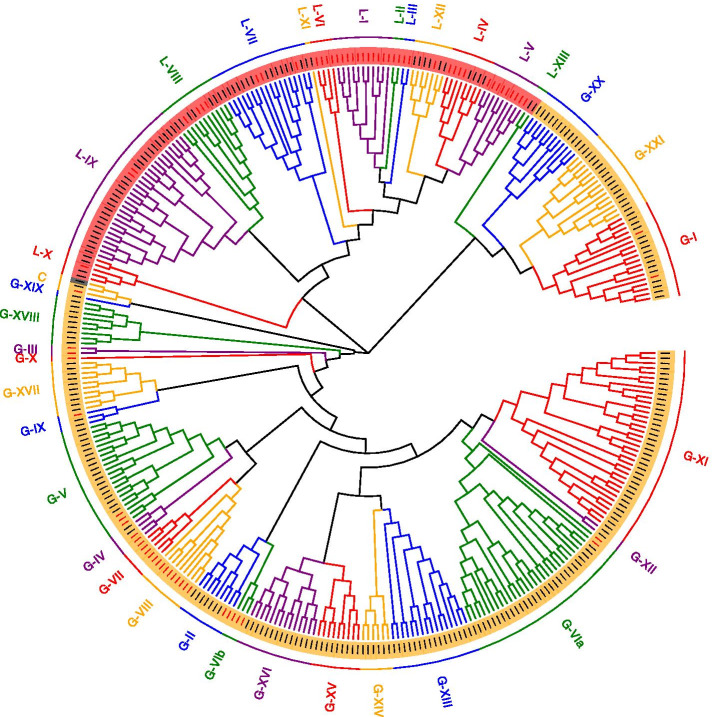
Phylogenetic analysis and classification of peanut and *A. thaliana* LecRLK proteins. The phylogenetic tree was established with full sequences using the maximum-likelihood method with 1000 bootstrap replications and the evolutionary distances were computed using the p-distance method. Red sequences indicated the AtLecRLKs. Each RLK clade was depicted by a different colour, representing the 35 clades that were identified; labelled lines on the outside of the tree represent clade names as defined in the text

To explore the phylogenetic relationships within the AhRLK class, full-length amino acid sequences of LRR-RLKs and LecRLKs were analysed separately. AhLRR-RLKs and AhLecRLKs were clustered with AtLRR-RLKs (209) and AtLecRLKs (76) respectively. The RLK classification in Arabidopsis was followed to analyse the phylogenetic relationship of peanut RLKs. AhLRR-RLKs were divided into 24 subclades in the ML tree (Fig. 1). The largest subclade LRR-XI contains 74 members, while the smallest subclade LRR-V contains only 1 member. Following the classification standards of Marcella [24] and Klass [25], peanut LecRLKs were classified into 35 subfamilies and subdivided into 3 classes: C-type LecRLKs (C-LecRLKs), L-type LecRLKs (L-LecRLKs) and G-type LecRLKs (G-LecRLKs) (Fig. 2). The largest subclades G-LecRLKs-XI and L-LecRLKs-IX contains 37 and 28 members separately, while no members from G-LecRLKs-VIb, G-LecRLKs-VIII, G-LecRLKs-VII, G-LecRLKs-X, G-LecRLKs-III, L-LecRLKs-VI, L-LecRLKs-I, L-LecRLKs-II, L-LecRLKs-III, and L-LecRLKs-V were found in the peanut.

### Chromosomal location and gene duplication of *AhRLK*s

**Fig. 3:**
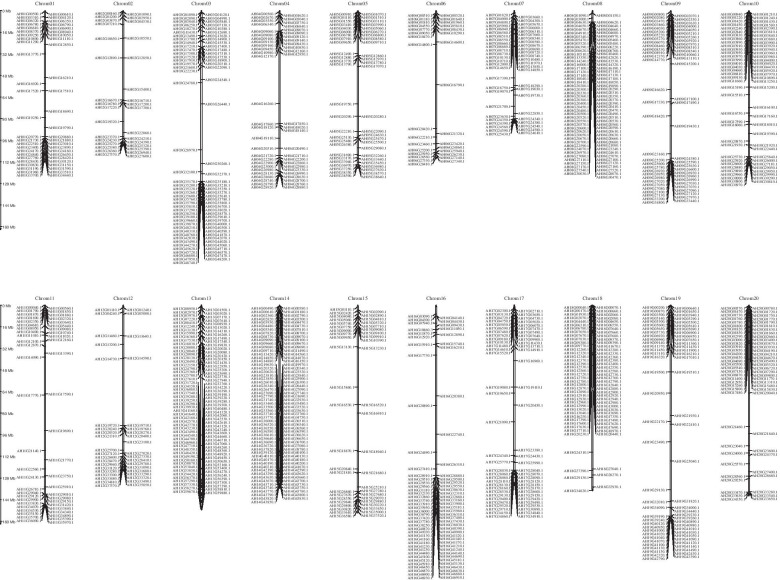
Genomic distribution of *AhRLK*s across peanut chromosomes. Chromosomal locations of *AhRLK*s are indicated based on the physical position of each gene. The positions of genes on each chromosome were drawn with MG2C (Map Gene 2 Chromosome v2) software and the number of chromosomes was labelled on the top of each chromosome

Physical positions of *AhRLK*s obtained from the “Peanut Genome resource” (http://peanutgr.fafu.edu.cn/) [26] were used to map them onto peanut chromosomes. Chromosome location information demonstrated that all the *AhRLK*s were unevenly distributed among the 20 chromosomes of the peanut, and 1.14% (15/1311) did not show assembly information (Fig. 3). Many *AhRLK*s were located on chromosomes 14 (111, 8.47%) and 13 (106, 8.09%), while only 31 (2.36%) *AhRLK*s were located on chromosome 6. Regarding LRR-RLKs, subfamilies LRR-XI and LRR-III were present on all chromosomes, while others were found only on some chromosomes. The majority of the LRR-RLKs and LecRLKs were located on chr 3, 13, 8 and 18 (Additional file 3), in particular, all members of the G-LecRLKs-XVII and G-LecRLKs-VIa subfamilies were distributed on chr 8 and 18 (Additional file 4, Fig. 4).

**Fig. 4:**
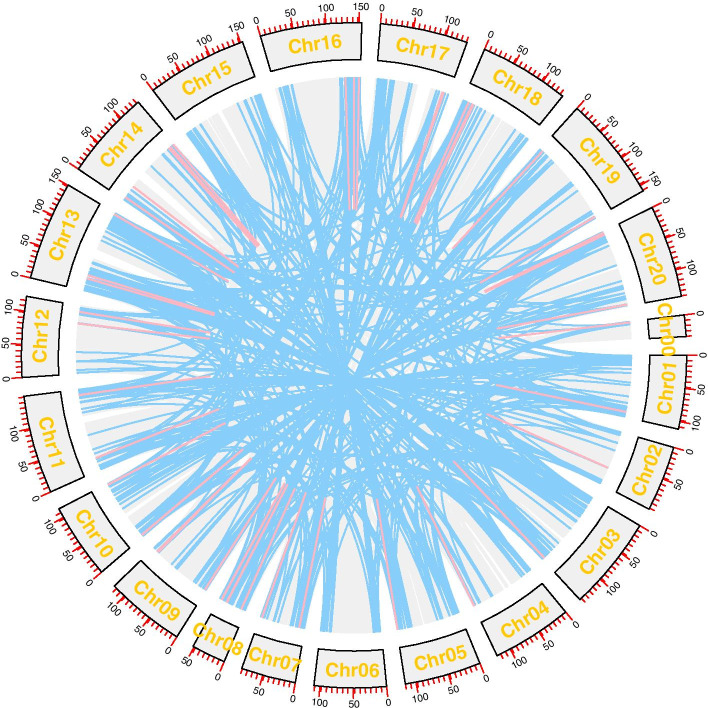
Schematic representations of the interchromosomal relationships of the *AhRLK*s. The red lines indicate tandem duplicated gene pairs; the blue lines indicate segmented duplicated gene pairs

**Fig. 5:**
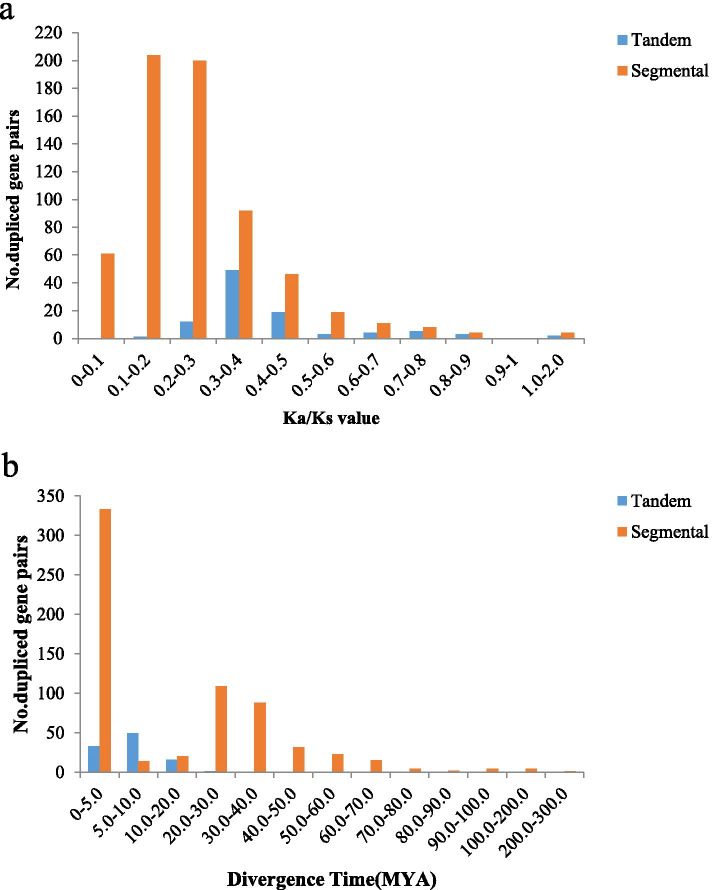
The distribution of *Ka/Ks* values and divergence time (MYA) in all tandem and segmental duplicated *AhRLK*s. **a** The distribution of *Ka/Ks* values in all tandem and segmental duplicated *AhRLK*s. **b** The distribution of divergence time (MYA) in all tandem and segmental duplicated *AhRLK*s

Gene replication events play an important role in the evolution of new functions of proteins and the expansion of genomes. Segmental duplication and tandem duplication are the main causes of the expansion of gene families in plants [27]. The position of two or more *AhRLK*s on the chromosome within 100 kb was considered a tandem duplication cluster. The results showed that approximately 9.53% (125/1311) of the genes were located in tandem duplication regions and constituted 52 clusters (Additional file 5). Among these genes, 5.66% (31/548) of AhLRR-RLKs and 17.51% (48/274) of AhLecRLKs were located in regions with tandem duplications. The largest tandem duplication cluster contained five genes, while the smallest cluster contained only two. Approximately 61.78% (810/1311) of the gene (810/1311) genes were located in segmental duplication regions. Up to 66.60% (365/548) of AhLRR-RLKs and 37.96% (104/274) of AhLecRLKs were located in regions with segmental duplications. To investigate the selection forces acting upon individual *AhRLK*s, the ratio of the nonsynonymous substitution rate to the synonymous substitution rate (Ka/Ks) was calculated. Among the 99 tandem duplicated gene pairs, the Ka/Ks ratios of 96.97% (96/99) of the gene pairs were less than 1 and 2.02% (2/99) were more than 1. One tandem duplication gene pair could not calculate the Ka/Ks value. Among the 654 segmental duplication gene pairs, the Ka/Ks ratios of 646 pairs (98.78%) were less than 1, and 4 pairs (0.61%) were more than 1. For four segmental duplication gene pairs Ka/Ks values could not be calculated (Fig. 5). In addition, we calculated the divergence time with the formula T = Ks/2r, in which r is the rate of divergence for nuclear genes from plants. The r of dicotyledonous plants was taken to be 1.5*10^-8 synonymous substitutions per site per year according to the methods of Koch [28]. The results showed that 82.82% (82/99) of tandem duplication events occurred 0–10 MYA, and 72.78% (476/654) of segmental duplication events occurred from 0–30 MYA (Additional file 6). Gene conversions play an important role in the coevolution of duplicated genes. Among the 52 tandem duplication clusters, 19 (36.54%) clusters showed statistically significant gene conversion events (P < 0.05). A total of 28 gene transformation events occurred in 52 tandem duplication clusters. The tract length of gene conversion ranged size from 16 to 1771 bp (Additional file 7).

### Phylogenetic analysis of Al-responsive AhRLKs

**Fig. 6:**
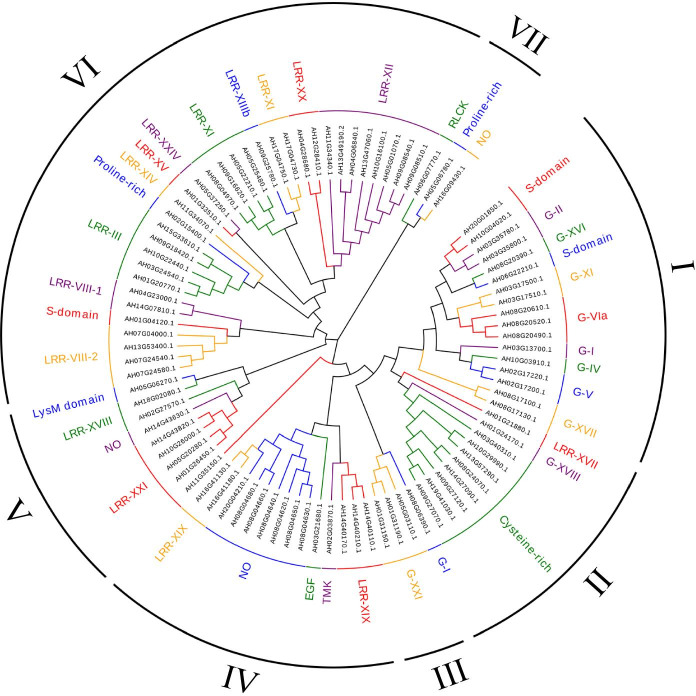
Phylogenetic analysis of Al-responsive *AhRLK*s. The full-length amino acid sequences of 90 Al-responsive *AhRLK*s were aligned by Clustal X, and the phylogenetic tree was constructed using MEGA 7. All Al-responsive *AhRLK*s were classified into 7 distinct groups based on the nomenclature of Arabidopsis LRR-RLKs and LecRLKs

In a previous study, we performed a transcriptome analysis to identify differentially expressed genes (DEGs) and pathways between two peanut cultivars under Al Stress [29]. In this study, we scrutinized transcriptome data to detect the AhRLKs involved in the Al response. Genes with log2-transformed ratio FPKM values greater than 1 or less than -1 were defined as differentially expressed genes. A total of 90 Al-responsive AhRLKs, including 44 LRR-RLKs, 19 LecRLKs, 8 cysteine-rich RLKs, 1 EGF-RLKs, 2 proline-rich RLKs, 4 s-domain RLKs, 1 TMK RLK, 1 RLCK RLK, 1 LysM domain RLK, and 9 no obvious domains (Additional file 2). To reveal the evolutionary relationships of these proteins, a phylogenetic tree was constructed using the ML method (Fig. 6). Phylogenetic analysis of all 90 AhRLKs revealed that the Al-responsive AhRLKs were further classified into 7 groups, including 48.9% LRR-RLKs, 21.1% LecRLKs and 8.9% CRKs. The phylogenetic tree showed that most of these genes belonged to LRR-RLKs and LecRLKs, covering the main subfamilies of LRR-RLKs and LecRLKs. Interestingly, these Al-responsive AhRLKs were evenly distributed across the LecRLK family, but unevenly distributed across the LRR-RLK families, focusing on LRR-III, LRR-XI, LRR-XII, LRR-VIII-1, and LRR-VIII-2.

### Characterization of the amino acid sequences and gene structure of Al stress-related *AhRLK*s

**Fig. 7:**
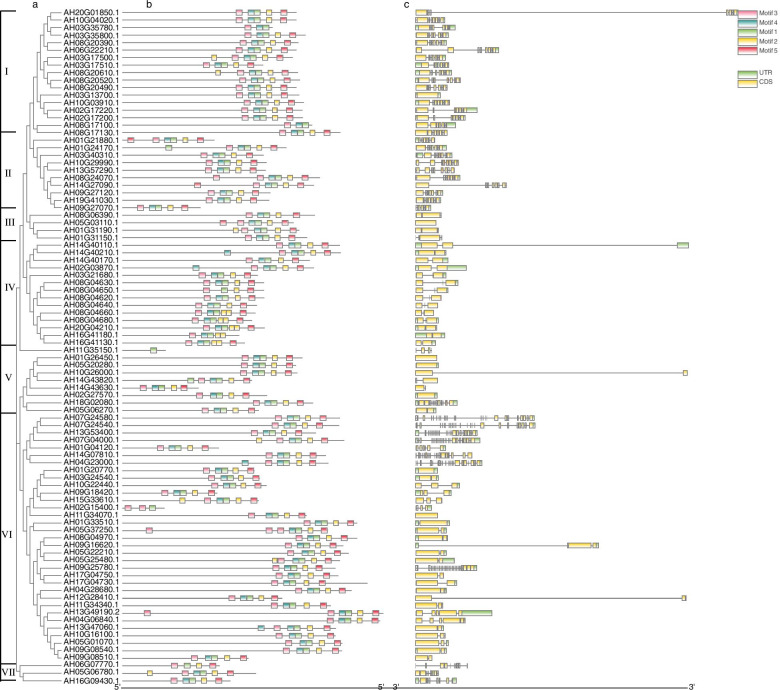
Phylogenetic relationships, gene structures and compositions of the conserved protein motifs of the Al-responsive *AhRLK*s. **a** The phylogenetic tree was constructed based on the full-length amino acid sequences of Al-responsive *AhRLK*s using MAGA 7. **b** Exon–intron structures of Al-responsive *AhRLK*s. Green boxes indicate untranslated 5'- and 3'-regions; yellow boxes indicate exons, and black lines indicate introns. **c** The motif compositions of the Al-responsive *AhRLK*s*.* The motifs, numbered 1–5, are displayed in different coloured boxes. The scale bar at the bottom indicates the base pair of genes

As shown in Fig. 7, 90 Al stress-related AhRLKs were divided into 7 groups. The diversification of exons/introns has been reported to be an important reason for the evolution of certain gene families [30]. The distribution of exons/introns of *AhRLK*s was further analysed. The results showed that 7.8% of Al stress-related *AhRLK*s (7/90) had no introns. One, two and three introns were found in 30% (27/90), 15.6% (14/90) and 1.1% (1/90) Al stress-related *AhRLK*s, respectively. Meanwhile, 45.6% (41/90) of the genes had more than three introns. All genes in subgroups I, II and VII contained more than three introns. Among these 30 genes, only one was LRR-RLK gene in subgroup II while 15 were LecRLKs in subgroups I and II (Fig. 6, Additional file 2).The majority of genes in subgroups III, IV and VI contained one or two introns, of which 70.6% (36/51) were LRR-RLKs, and 7.8% (4/51) were LecRLKs. This result was similar to the study in which most LRR-RLKs in Arabidopsis had fewer than three introns [31]. Moreover, to analyse the diversity of the Al stress-related AhRLKs, the MEME tool was used to predict putative motifs of these proteins. A total of 5 different motifs were detected in Al stress-related AhRLKs and named motifs 1 to 5 (Additional file 8). Genes in subgroup I 82.4% (14/17), 70% (7/10) of genes in subgroup II, 50% of genes in subgroup III, 42.9% (6/14) of genes in subgroup IV, 88.9% (8/9) of genes in subgroup V, 75.8% (25/33) of genes in subgroups VI, and 33.3% (1/3) of genes in subgroup VII were shown to contain the same motif composition as motif 3-motif 4-motif 1-motif 2-motif 5.

### Expression profiles of Al-responsive *AhRLK*s in different tissues

**Fig. 8:**
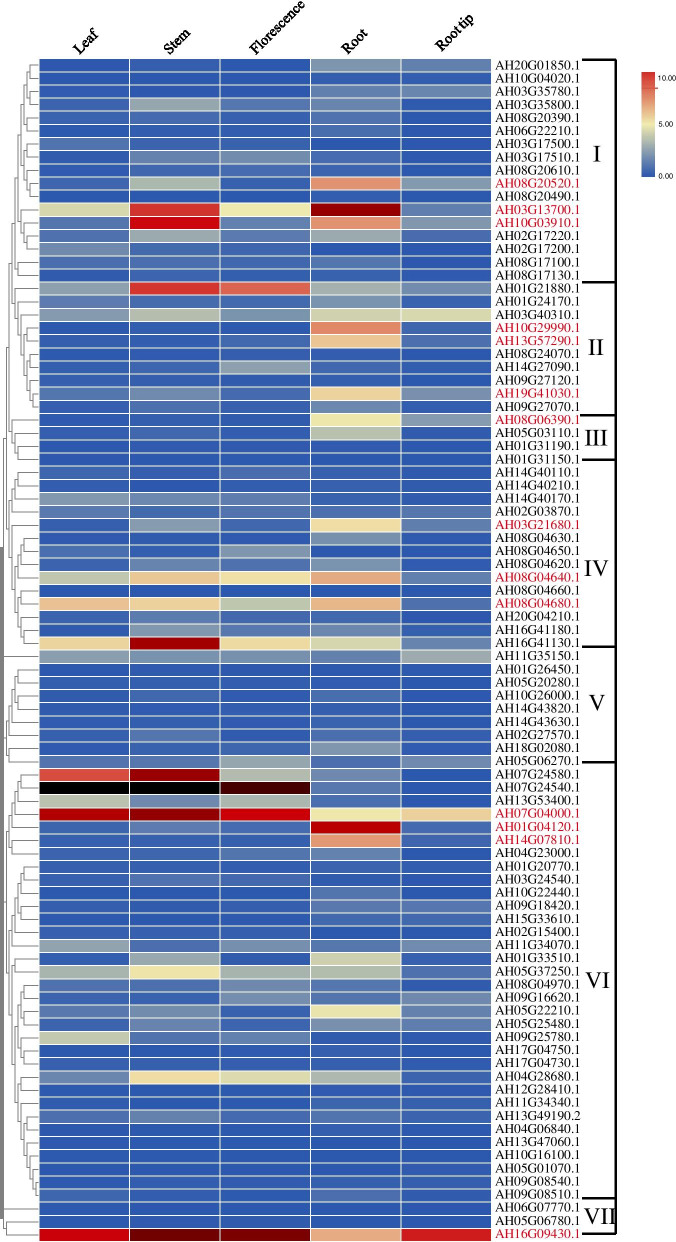
Expression profiles of Al-responsive *AhRLK*s in different tissues. FPKM values were used to create the heat map with clustering. The scale represents the relative signal intensity of FPKM values

To further understand the role of Al-responsive *AhRLK*s in peanut growth and development, the expression profiles of Al-responsive *AhRLK*s from different organs, including leaves, stems, florescence, roots and root tips, were tested in a cultivated variety (*A. hypogaea* L.) using transcriptomic data (Fig. 8). Among these Al-responsive *AhRLK*s, the majority (78/90, 86.7%) were expressed in all organs examined. Six genes (6.7% *AH16G41130.1*, *AH07G04000.1*, *AH07G24540.1*, *AH07G24580.1*, *AH08G04680.1*, and *AH16G09430.1*) were expressed at a high level (value > 5) in leaves, 12 genes (13.3% *AH05G37250.1*, *AH04G28680.1*, *AH16G41130.1*, *AH01G21880.1*, *AH07G04000.1*, *AH07G24540.1*, *AH07G24580.1*, *AH03G13700.1*, *AH10G03910.1*, *AH08G04680.1*, *AH08G04640.1*, and *AH16G09430.1*) in stems, 6 genes (6.7%, *AH16G41130.1*, *AH01G21880.1*, *AH07G04000.1*, *AH07G24540.1*, *AH08G04640.1*, and *AH16G09430*) in florescences, and 14 genes (15.6%, *AH07G04000.1*, *AH03G13700.1*, *AH10G03910.1*, *AH08G04680.1*, *AH08G04640.1*, *AH16G09430.1*, *AH14G07810.1*, *AH03G21680.1 AH19G41030.1*, *AH13G57290.1*, *AH10G29990.1*, *AH08G20520.1*, *AH08G06390.1*, and *AH01G04120.1*) in roots or root tips.

### Expression patterns of Al-responsive *AhRLK*s under Al stress

**Fig. 9:**
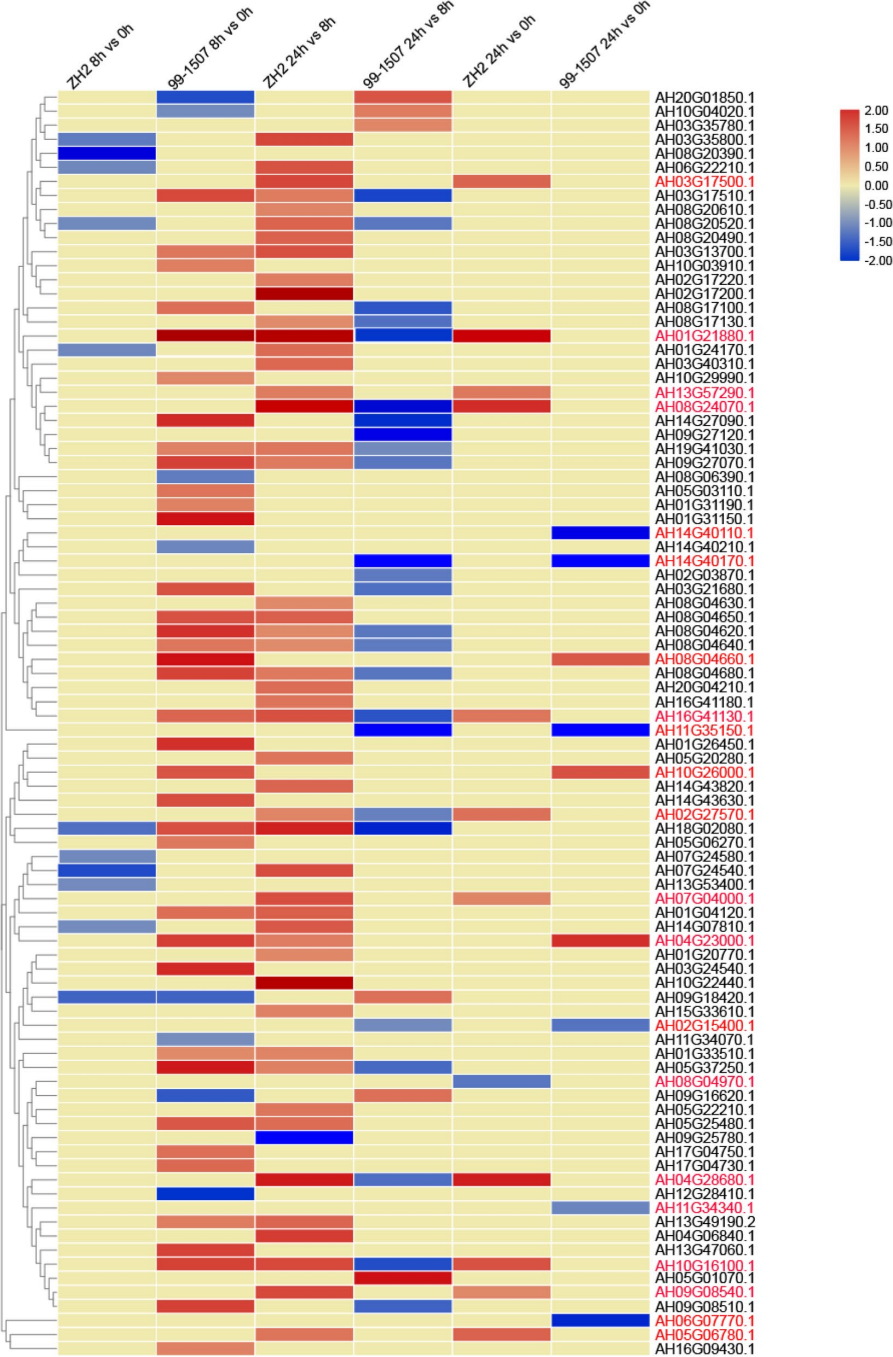
Expression profiles of Al-responsive *AhRLK*s in the two varieties. The RNA-seq data of each gene in peanut root tips under Al stress in the two cultivars are shown here. Heatmap showed the log2-transformed ratio FPKM values. The genes were on the right of the expression bar

To further investigate the putative functions of Al-responsive *AhRLK*s, an RNA-Seq dataset that was generated from different Al treatment time points were utilized to reveal the expression profiles of these genes under Al stress. The expression profiles of Al-responsive *AhRLK*s are shown in histograms (Fig. 9). As shown in Fig. 9, 41.1% (37/90) of *AhRLK*s exhibited > twofold upregulation under Al stress for 8 h in 99–1507. A total of 12.2% (11/90) and 8.9% (8/90) of *AhRLK*s exhibited > twofold down regulation under Al stress for 8 h in ZH2 and 99–1507, respectively. Among the *AhRLK*s, 3.3% (3/90) and 12.2% (11/90) exhibited > twofold up regulation in the 24 h vs 0 h Al-treatment comparison, 6.7% (6/90) and 1.1% (1/90) *AhRLK*s exhibited > twofold down regulation in 24 h vs 0 h Al-treatment comparison in the ZH2 and 99–1507, respectively (Additional file 9).

## Discussion

### Segmental duplication events played an important role in AhRLK family evolution

RLKs are involved in a variety of plant physiological processes and various abiotic and biotic stress responses [32, 33]. In this study, a total of 1311 AhRLKs, including 548 LRR-RLKs, 274 LecRLKs, 83 cysteine-rich RLKs, 76 EGF-RLKs, 49 proline-rich RLKs, 46 s*-domain RLK*s, 22 *TMK-RLK*s, 2 *TNFR-RLK*s, 1 RRO-RICH RLK, 28 RLCK-RLKs, 24 LysM-RLKs, and 158 no obvious domain RLKs, were identified from whole peanut genome sequences (Additional file 1).

The 548 LRR-RLKs were classified into 24 subfamilies (I to XXIV) based on their phylogenetic relationship with Arabidopsis, which was 2 times the number of Arabidopsis LRR-RLKs (Fig. 1). In general, the number of LRR-RLKs for most of the subfamilies among the peanut was two times the number of LRR-RLKs of Arabidopsis, except LRR-XII, LRR-XIV, LRR-XV and LRR-XVI, which had more than three times the number of members of Arabidopsis. Only one subfamily, LRR-V, had fewer members than Arabidopsis. The number of LecRLKs was over 3 times the number of AtLecRLKs (Fig. 2). The subfamilies in the peanut such as L-LecRLK-VII, L-LecRLKs-IX and G-LecRLKs-VIa were much larger than the subfamilies in Arabidopsis, while some subfamilies, including G-LecRLKs-VIb, G-LecRLKs-VIII, G-LecRLKs-VII, G-LecRLKs-X, G-LecRLKs-III, L-LecRLKs-VI, L-LecRLKs-I, L-LecRLKs-II, L-LecRLKs-III and L-LecRLKs-V, were not found in the peanut (Tables 1 and 2). Polyploidy may cause an increase in the number of genome genes in the peanut. In recent research, a total of 309, 379, 467, 531, and 543 LRR-RLKs have been identified in diploid rice [34], diploid poplar [35], tetraploid soybean [36], allohexaploid wheat [37] and tetraploid cotton [38], respectively, indicating that larger gene families are present in polyploid plants. Other factors, including tandem duplication, segmental duplication, and exon duplication and shuffling, also contribute to the expansion of gene families.

**Table 1:** Total number of receptors distributed in the different subfamilies of LRR-RLKs

Subfamilies	Plant species	
	Peanut	*A. thaliana*
**LRR-I**	34	38
**LRR-II**	27	13
**LRR-III**	70	41
**LRR-IV**	10	4
**LRR-V**	1	9
**LRR-VI-1**	17	6
**LRR-VI-2**	9	4
**LRR-VII**	9	8
**LRR-VIII-1**	18	7
**LRR-VIII-2**	32	12
**LRR-IX**	3	4
**LRR-X-a**	11	4
**LRR-X-b**	6	9
**LRR-XI**	74	29
**LRR-XII**	61	9
**LRR-XIII-a**	7	3
**LRR-XIII-b**	4	3
**LRR-XIV**	10	3
**LRR-XV**	6	2
**LRR-XVI**	5	1
**LRR-XVII**	65	0
**LRR-XVIII**	6	0
**1RR-XIX**	15	0
**LRR-XX**	32	0
**LRR-XXI**	2	0
**LRR-XXII**	2	0
**LRR-XXIII**	2	0
**LRR-XXIV**	10	0
**Total**	548	209

**Table 2:** Total number of receptors distributed in the different subfamilies of LecRLKs

Subfamilies	Plant species	
	Peanut	*A. thaliana*
**G-LecRKs-I**	16	2
**G-LecRKs-II**	7	2
**G-LecRKs-III**	0	2
**G-LecRKs-IV**	2	2
**G-LecRKs-V**	18	3
**G-LecRKs-VIa**	29	2
**G-LecRKs-VIb**	0	3
**G-LecRKs-VII**	0	5
**G-LecRKs-VIII**	0	9
**G-LecRKs-IX**	2	1
**G-LecRKs-X**	0	1
**G-LecRKs-XI**	37	0
**G-LecRKs-XII**	2	0
**G-LecRKs-XIII**	16	0
**G-LecRKs-XIV**	6	0
**G-LecRKs-XV**	9	0
**G-LecRKs-XVI**	14	0
**G-LecRKs-XVII**	10	0
**G-LecRKs-XVIII**	9	0
**G-LecRKs-XIX**	1	0
**G-LecRKs-XX**	12	0
**G-LecRKs-XXI**	15	0
**L-LecRKs-I**	0	11
**L-LecRKs-II**	0	2
**L-LecRKs-III**	0	2
**L-LecRKs-IV**	4	4
**L-LecRKs-V**	0	9
**L-LecRKs-VI**	0	4
**L-LecRKs-VII**	15	3
**L-LecRKs-VIII**	7	4
**L-LecRKs-IX**	28	2
**L-LecRKs-X**	4	1
**L-LecRKs-XI**	1	0
**L-LecRKs-XII**	6	1
**L-LecRKs-XIII**	2	0
**C-LecRKs**	2	1
**Total**	274	76

Gene duplication was the main mechanism for evolutionary events [39]. The gene duplication results revealed that 9.53% (125/1311) of *AhRLK*s were located in regions with tandem duplications, and 61.78% (810/1311) were located in regions with segmental duplications, which indicated that segmental duplication played a major role in the evolution of *AhRLK*s (Additional file 5). Among the AhLRR-RLKs 5.66% (31/548) and 66.60% (365/548) were found to be located in the tandem duplication region and the segmental duplication region, respectively. This finding is consistent with the work in soybean that segmental duplication may be the main mechanism of LRR-RLK amplification [36]. In addition, the ka/ks ratios of 94.9% (1290/1360) of *AhRLK*s were less than 1, which suggested that most *AhRLK*s were selected for purification (Fig. 5). The ka/ks ratios of six gene pairs including, AH16G29500.1 and AH16G29530.1, AH16G29500.1 and AH16G29560.1, AH08G17100.1 and AH18G07640.1, AH03G13490.1 and AH13G15990.1, AH08G05340.1 and AH17G29130.1 and AH14G36690.1 and AH14G43630.1 were more than 1, which indicated that these genes were in a state of positive selection in peanuts, evolving rapidly, and might be very important for the evolution of the peanut. We also calculated the divergence time, and the results showed that many tandem duplication events appeared to have occurred during relatively recent key periods 0–10 MYA, and many segmental duplication events appeared to have occurred during 0–30 MYA (Fig. 5b; Additional file 6), illustrating that these *AhRLK*s were generated by recent gene duplication events in *Arachis hypogaea* L. Moreover, 28 gene transformation events were detected among the genes in the 52 tandem duplication clusters, and 44 genes involved in at least one gene conversion event, which suggested that gene conversion events had taken place between the duplicated *AhRLK*s. Gene conversion is implicated in the concerted evolution of multigene families, which helps gene evolution by allowing more time for duplicated genes to obtain selectable differences [40, 41]. As changes in expression patterns are an important factor that cause genes to gain selectable differences [40, 42], studying the temporal and spatial expression patterns of these genes would be of interest.

### Conservation of the AhRLKs in response to Al stress

In this study, a total of 90 AhRLKs were identified as Al stress-related genes, which were divided into 7 groups (Fig. 7). Most of the subgroups show certain regularity of exon–intron structure. For instance, all genes in subgroups I, II and VII contained more than three introns. Members belonging to the same subgroup had similar exon/intron organization. Furthermore, 5 conserved motifs were identified in these AhRLKs and the motif compositions among subgroups were consistent with the phylogenetic classification. These results indicated that the members in the subgroups were more conservative in the evolution.

### Diversity roles of Al-responsive *AhRLK*s in different subgroups

To further understand the Al-responsive *AhRLK*s in the peanut, we investigated the potential functions of each subgroup (Table 3). In subgroup I, *PERK1* has been reported to regulate ABA signalling pathways and modulate the expression of genes related to cell elongation and ABA signalling during root growth [43], implying that the genes in Subgroup I were essential to plant signalling and growth. The inhibition of root elongation is known to be the primary symptom of Al toxicity, and the members of subgroup I may take part in the Al response by influencing cell elongation. The genes known to function in subgroup II were reported to play a role in plant signal transduction, plant growth and biotic stress response, for instance, *PXC1* and *CRCK1* played a role in signal transduction [44, 45], *PRK1* was essential for the postmeiotic development of pollen [46], *FLS2* was involved in preinvasive immunity against bacterial infection [47], and *RCH1* was critical to the resistance of the hemibiotrophic fungal pathogen *Colletotrichum higginsinaum* [48]. In Subgroup III, *ANXUR1/ANXUR2* were involved in controlling pollen tube rupture during the fertilization process and regulating signal transduction [49]. *FERONIA* was required for cell elongation during vegetative growth [50], suggesting that the genes in subgroup III might play an important role in plant morphology. In subgroup IV, *TMK1* was an essential enzyme for DNA synthesis in bacteria [51], which indicated that the genes of subgroup IV might play a critical role in cell expansion and proliferation regulation. The subgroup V gene *RLK1* was reported to increase tolerance to salinity, heavy metal stresses, and *Botrytis cinerea* infection [52], suggesting that the genes of subgroup V are implicated in biotic and abiotic stress responses. In subgroup VI, *CRK5* was reported to respond to drought and salt stresses [53], and *CRK45* was a potentially positive regulator of ABA signalling in early seedling growth [54] and stomatal movement [55], indicating that the genes of subgroup VI are critical to the abiotic stress response and related to plant morphology. The reported genes in subgroup VII, such as *GsSRK*, were shown to be positive regulators of plant tolerance to salt stress [56], and *SD1-29* improved plant resistance to bacteria [57], showing that the genes of subgroup VII have critical roles in the response to biotic and abiotic stresses. In general, Al-responsive *AhRLK*s in different subgroups take part in the Al response by different pathways. Subgroups I and II are related to signal transduction, subgroup II is implicated in the biotic stress response, subgroups III and VI play an essential role in plant morphology, subgroup IV plays a critical role in cell expansion and proliferation regulation, and subgroups V and VII are critical to the biotic stress and abiotic stress response (Table 3).

**Table 3:** The classification of subgroups for Al responsive *AhRLK*s

Subgroups	Gene ID	Gene Name	Reported	Function
I	*AH05G06780.1*	Proline-rich receptor-like protein kinase PERK4	PERK1	responses to wounding and treatment with salicylic acid and PERK1 mRNA accumulation in response to these treatments shows a role in plant defense signaling [43]
II	*AH09G18420.1*	Leucine-rich repeat receptor-like protein kinase PXC1	PXC1	a regulator of secondary wall formation correlated with the TDIF-PXY/TDR-WOX4 signaling pathway [44]
II	*AH01G04120.1*	Calmodulin-binding receptor-like cytoplasmic kinase 1	CRCK1	plays a role in stress signal transduction in plants [45]
II	*AH13G53400.1*	Probable LRR receptor-like serine/threonine-protein kinase RKF3	RKF1	regulates early flower primordia during stamen development [58]
II	*AH13G49190.2* *AH04G06840.1*	LRR receptor-like serine/threonine-protein kinase FLS2	FLS2	involves in preinvasive immunity against bacterial infection [59]
II	*AH02G15400.1*	Proline-rich receptor-like protein kinase PERK3	PERK1	responses to wounding and treatment with salicylic acid and PERK1 mRNA accumulation in response to these treatments shows a role in plant defense signaling [43]
II	*AH01G20770.1* *AH03G24540.1*	Pollen receptor-like kinase 3	PRK1	PRK1 is essential for postmeiotic development of pollen [46]
II	*AH09G25780.1*	LRR receptor-like serine/threonine-protein kinase ERL1	ERECTA	regulates elongation of above-ground organs [60]
II	*AH08G04970.1*	LRR receptor-like serine/threonine-protein kinase RCH1	RCH1	resistances to the hemibiotrophic fungal pathogen colletotrichum higginsianum [48]
II	*AH09G16620.1*	Leucine-rich repeat receptor-like protein kinase PXL1	PXL1	regulates signal transduction pathways under temperature fluctuations [61]
II	*AH05G37250.1*	Leucine-rich repeat receptor-like tyrosine-protein kinase PXC3	PXC1	a regulator of secondary wall formation correlated with the TDIF-PXY/TDR-WOX4 signaling pathway [44]
II	*AH05G22210.1*	LRR receptor-like serine/threonine-protein kinase HSL2	HSL2	involves in Floral organ abscission and lateral root emergence [62]
II	*AH05G25480.1*	Receptor-like protein kinase HSL1	HSL1	participates in the Repression of Seed Maturation Genes in Arabidopsis Seedlings [63]
II	*AH02G27570.1*	Probable LRR receptor-like serine/threonine-protein kinase RKF3	RKF1	regulates early flower primordia during stamen development [58]
III	*AH01G26450.1*	Receptor-like protein kinase ANXUR1	ANXUR1/ANXUR2	control pollen tube rupture during the fertilization process in *A. thaliana* [49]
III	*AH10G26000.1* *AH14G43820.1* *AH05G20280.1*	Receptor-like protein kinase FERONIA	FERONIA	affects plant reproduction, development, and stress tolerance [50]
III	*AH05G06270.1*	LysM domain receptor-like kinase 4	RLK1	activates defense and Abiotic-Stress Responses [52]
III	*AH14G43630.1*	Receptor-like protein kinase ANXUR2	ANXUR1/ANXUR2	control pollen tube rupture during the fertilization process in Arabidopsis thaliana [49]
III	*AH11G35150.1*	LRR receptor-like serine/threonine-protein kinase HSL2	HSL2	involved in Floral organ abscission and lateral root emergence [62]
IV	*AH02G03870.1*	Receptor protein kinase TMK1	TMK1	an essential enzyme for DNA synthesis in bacteria, phosphorylating deoxythymidine monophosphate (dTMP) to deoxythymidine diphosphate (dTDP), and thus is a potential new antibacterial drug target [51]
V	*AH01G31190.1* *AH01G31150.1*	G-type lectin S-receptor-like serine/threonine-protein kinase RLK1 isoform X2	RLK1	activates defense and Abiotic-Stress Responses [52]
VI	*AH09G27120.1* *AH19G41030.1*	Cysteine-rich receptor-like protein kinase 29	CRK45/CRK5	response to abscisic acid and abiotic stressesa potentially positive regulator of ABA signaling in early seedling growth, stomatal movement and plant drought tolerance [53, 54]
VI	*AH08G24070.1* *AH14G27090.1*	Cysteine-rich receptor-like protein kinase 25	CRK45/CRK5	response to abscisic acid and abiotic stresses, a potentially positive regulator of ABA signaling in early seedling growth, stomatal movement and plant drought tolerance[53, 54]
VI	*AH10G29990.1* *AH13G57290.1* *AH09G27070.1*	Cysteine-rich receptor-like protein kinase 10	CRK45/CRK5	response to abscisic acid and abiotic stresses, a potentially positive regulator of ABA signaling in early seedling growth, stomatal movement and plant drought tolerance[53, 54]
VI	*AH03G40310.1*	Cysteine-rich receptor-like protein kinase 2	CRK45/CRK5	response to abscisic acid and abiotic stresses, a potentially positive regulator of ABA signaling in early seedling growth, stomatal movement and plant drought tolerance[53, 54]
VII	*AH10G03910.1*	G-type lectin S-receptor-like serine/threonine-protein kinase B120	GsSRK	a positive regulator of plant tolerance to salt stress [56]
VII	*AH20G01850.1* *AH10G04020.1* *AH06G22210.1*	Receptor-like serine/threonine-protein kinase SD1-8	SD1-29	resistances to bacteria in crop species [64]
VII	*AH01G24170.1*	G-type lectin S-receptor-like serine/threonine-protein kinase B120	GsSRK	a positive regulator of plant tolerance to salt stress [56]

Note: only the Al responsive *AhRLK*s with characterized homologs were listed in the table

The *AtRLK* gene family plays a role in plant growth and development processes [63]. As shown in the histograms in Fig. 8, the expression pattern of the Al-responsive *AhRLK*s exhibited tissue specificity, and approximately 2.2% (2/90, *AH07G04000.1* and *AH16G09430.1*) of Al-responsive *AhRLK*s were expressed in all four tested organs with high expression levels (value > 5) in the peanut, implying that these genes might play essential roles in plant growth and development. Approximately 2.2% (2/90, *AH16G41130.1* and *AH07G24540.1*) of Al-responsive *AhRLK*s were expressed specifically and at a high level in aerial organs. About 8.8% (8/90, *AH14G07810.1, AH03G21680.1 AH19G41030.1 AH13G57290.1, AH10G29990.1, AH08G20520.1, AH08G06390.1,* and *AH01G04120.1*) of Al-responsive *AhRLK*s were expressed specifically and at a high level in roots or root tips. The tissue specificity of these Al-responsive *AhRLK*s indicates their key roles in tissue development or tissue functions. Additionally, 6 tissue nonspecific genes (*AH07G04000.1*, *AH03G13700.1*, *AH10G03910.1*, *AH08G04680.1*, *AH08G04640.1*, and *AH16G09430.1*) that were expressed at a high level specifically in roots are also worth considering. As shown in the histograms in Fig. 9, the majority of the Al-responsive *RLK*s were upregulated after 8 h of Al treatment in 99–1507, while only moderate changes were detected in some Al-responsive *RLK*s in ZH2, which suggested that Al-responsive *RLK*s responded rapidly to Al stress in the Al-tolerant variety. Although the genes had different expression profiles under Al stress in different varieties, the expression levels of 12 genes (*AH04G28680.1*, *AH16G41130.1*, *AH01G21880.1*, *AH10G16100.1*, *AH08G24070.1*, *AH02G27570.1*, *AH07G04000.1*, *AH09G08540.1*, *AH13G57290.1*, *AH03G17500.1*, *AH05G06780.1*, and *AH08G04970.1*) and 9 genes (*AH04G23000.1*, *AH11G34340.1*, *AH06G07770.1*, *AH14G40110.1*, *AH10G26000.1*, *AH02G15400.1*, *AH11G35150.1*, *AH14G40170.1*, *AH08G04660.1*), which reached their peak in 24 h vs 0 h Al-treatment comparison in ZH2 and 99–1507, implying important roles in Al stress responses. Among them, *AH01G21880.1* and *AH04G28680.1* were expressed at a high level in stems, implying their potential roles in regulating the growth of stems. *AH13G57290.1* was expressed specifically and at a high level in roots, implying its critical roles in mediating the Al response in peanut. *AH07G04000.1* was expressed in all four tested organs with high expression levels, and it might play essential roles in plant growth and development under Al stress. Taken together, our results revealed that 13 genes (*AH11G35150.1*, *AH08G24070.1*, *AH13G57290.1*, *AH02G27570.1*, *AH05G06780.1*, *AH02G15400.1*, *AH01G35150.1, AH14G27090.1,* AH05G37250.1, AH10G03910.1, *AH19G41030.1*, *AH10G29990.1,* and *AH10G26000.1*), whose homologues have been reported to be involved in early seedling growth regulation, early flower primordia and stamen development, lateral root emergence, abiotic stress responses and plant defence signalling in *Arabidopsis thaliana,* were important Al-responsive genes that may be suitable candidates for interpreting the mechanisms underlying the Al response in peanuts in future work.

## Conclusions

In this study, a total of 1311 RLKs were identified in the peanut genome, 2 times the number of Arabidopsis RLKs, including 548 LRR-RLKs and 274 LecRLKs. LRR-RLK represented the largest RLK gene family identified in plants. These *AhRLK*s were unevenly distributed among 20 chromosomes of peanut. Compared with tandem duplication, segmental duplication might play a more critical role in some *AhRLK*s. Furthermore, we identified a total of 90 Al-responsive *AhRLK*s by mining the transcriptome database. The exon/intron compositions and motif arrangements were considerably conserved among members in the same groups or subgroups. Analysis of transcriptome data revealed tissue expression patterns of the 90 Al-responsive *AhRLK*s, and tissue-specific expression genes were found. Among them, root-specific genes might play a key role in Al sensing and response in the peanut. The close phylogenetic relationship of Al-responsive AhRLKs and characterized AhRLKs in the same subgroup provided insight into their putative functions. Overall, this systematic analysis provided valuable information to understand the biological functions of the AhRLK genes under Al stress in peanut.

## Methods

### The resources of peanut AhRLKs

All RLK full-length amino acid sequences in Arabidopsis were downloaded from UniProt (https://www.uniprot.org/) and these sequences were used as queries to perform a BLASTP search against *A. duranensis* RLKs by NCBI (https://www.ncbi.nlm.nih.gov/). These resulting sequences were then used as new queries to conduct a BLASTP search again in PEANUT GENOME RESOURSE (http://peanutgr.fafu.edu.cn/), to avoid missing potential members. The redundant entries were removed manually. Then the resulting unique sequences were analysed with both SMART (http://smart.embl-heidelberg.de) [65] and NCBI’s Conserved Domains Database (CDD; http://www.ncbi.nlm.nih.gov/Structure/cdd/wrpsb.cgi) to ensure the presence of the RLK domains in newly identified members. Only proteins containing at least one kinase domain were considered putative AhRLKs*,* and 1311 AhRLKs were finally obtained. The amino acid residue base, and molecular weight were predicted with ExPaSy ProtParam tool (https://web.expasy.org/protparam/). The genome sequence, protein sequences and genome annotation of the peanut were performed according to PEANUT GENOME RESOURSE (http://peanutgr.fafu.edu.cn/).

### Multiple sequence alignments and phylogenetic tree construction of *AhRLK*s

The full-length amino acid sequences of LRR-AhRLKs, LecRLKs and 90 Al-responsive AhRLKs defined in the previous section were aligned using ClustalX in MEGA 7 with default parameters [66]. The phylogenetic tree based on the multiple sequence alignments of peanut LRR-RLKs (Fig. 1), LecRLKs (Fig. 2) and 90 AhRLKs in response to Al stress (Fig. 6) was generated by MEGA 7. A Poisson correction model was used to account for multiple substitutions, while alignment gaps were removed with partial deletion. The statistical strength was estimated by bootstrap resampling using 1000 replicates. Based on the multiple sequence alignment and the previously reported classification of *Arabidopsis thaliana*, the peanut *RLK*s were assigned to different subfamilies and subgroups [24, 67].

### Chromosomal locations and duplication analysis for peanut *RLK*s

The physical location of *AhRLK*s on the chromosomes was obtained from the PEANUT GENOME RESOURSE database (http://peanutgr.fafu.edu.cn/). All members of *AhRLK*s were mapped onto peanut chromosomes based on their physical positions, and chromosomal location images were produced with the online software Map Gene 2 Chromosome v2 (MG2C:http://mg2c.iask.in/mg2c_v2.0/). The chromosome location information of the peanut was extracted from GFF files that contain the information of peanut genome annotation. BLASTP was performed to search for potential homologous gene pairs (E-value < 1e^−5^) across genomes. Information on homologous pairs was used as input to identify syntenic chains by MCScanX [68]. In addition, MCScanX was also used to identify tandem and segmental duplications in the *AhRLK* gene family. *RLK*s clustered together within 100 kb were regarded as tandem duplicated genes based on the criteria of other plants. The diagram was generated by TBtools [69]. The nonsynonymous (Ka) and synonymous (Ks) substitution ratios were calculated by Simple Ka/Ks Caculator in TBtools. The divergence time was calculated with the formula T = Ks/2r, and the r of dicotyledonous plants was 1.5*10^-8 synonymous substitutions per site per year [70]. We used the Geneconv program with default parameters to search evidence for tandem duplication cluster gene conversion (http://www.math.wustl.edu/~sawyer/geneconv/) [71]. Since GENECONV required at least three sequences for detecting gene conversion events, tandem duplication clusters that contained at least 3 genes were detected. For this program, the clustalW (CDS) alignment was used as the input. Geneconv can detect candidate fragments of directed gene conversion between gene pairs (allowing mismatch). Gene conversion events were considered as statistically significant when *P* < 0.05.

### Gene structure and motif analysis of *AhRLK*s in response to Al stress

The exon–intron structures of 90 peanut Al-responsive AhRLKs were determined based on their coding sequence alignments and their respective genomics sequences, while diagrams were obtained from the online program Gene Structure Display Server with default parameters (http://gsds.cbi.pku.edu.cn/) [72]. To identify the conserved motifs of the Al response AhRLKs, the MEME (Multiple Em for Motif Elicitation) tool was used to predict putative motifs of these proteins (http://meme-suite.org/) [73]. The combination of phylogenetic tree, gene and protein structures was generated using TBtools.

### Expression Pattern Analysis for Al-responsive *AhRLK*s

By scrutinizing the existing transcriptome data, the expression profiles of Al-responsive *AhRLK*s in different tissues under normal conditions and in the root tips of different peanut varieties under Al stress were analysed. The raw RNA-seq reads in five tissues, including leaf, stem, florescence, root and root tips, were available at Peanut Genome Resource (http://peanutgr.fafu.edu.cn/). The RNA-seq data of ZH2 (ZhongHua No.2, Al sensitive) and 99–1507 (Al tolerant) under Al treatment were deposited in the database of the National Center for Biotechnology Information (NCBI) under accession number PRJNA525247 (https://www.ncbi.nlm.nih.gov/sra/PRJNA525247). Heat maps of the Log2-transformation ratio of FPKM values and gene FPKM values in Al-responsive *AhRLK*s of different varieties or tissues were visualized using TBtools.

## Supplementary Information

**Additional file 1:** Complete list and classification of 1311 *AhRLK*s in peanut.

**Additional file 2:** Complete list and classification of Al response *AhRLK*s in peanut.

**Additional file 3:** Subfamily and chromosome distribution of AhLRR-RLKs in peanut.

**Additional file 4:** Subfamily and chromosome distribution of AhLecRLKs in peanut.

**Additional file 5:** Tandem duplication clusters of *AhRLK*s.

**Additional file 6:** Devergence time among *AhRLK*s tandem duplication pairs and segmental duplication pairs.

**Additional file 7: **Tandom duplicated genes analyzed for gene conversion.

**Additional file 8: **The motif of Al stress-related *AhRLK*s.

**Additional file 9:** Expression Profiles of Al-responsive *AhRLK*s under Al stress.

## Data Availability

The datasets generated and analyzed during the current study are available from the corresponding author on reasonable request. The two peanut cultivars that had used to generate the RNA-seq data with Al treatment were kindly provided by Prof. Bo-shou Liao from the Oil Crop Research Institute, Chinese Academy of Agricultural Sciences (CAAS), and they were routinely planted on the farm of Guangxi University in Nanning, Guangxi Province, China and identified by Prof. He. In detail, ZhongHua No. 2 (ZH2) (85–007, CHINA PEANUNT DATA CENTEI, http://www.peanutdata.cn/variety/index.htm) has been used widely in agriculture practice while 99–1507 has not been approved for commercial use. The RNA-seq data of ZH2 and 99–1507 under Al treatment had been deposited in the database of the National Center for Biotechnology Information (NCBI) under accession number PRJNA525247 (https://www.ncbi.nlm.nih.gov/sra/PRJNA525247). The raw RNA-seq reads in different tissues and *AhRLK*s sequences are available at Peanut Genome Resource (http://peanutgr.fafu.edu.cn/).

## Abbreviations

*Ah*: *Arachis**hypogaea*.L
Al: Aluminum
*At*: *Arabidopsis thaliana*
EGF-RLK: Epidermal growth factor like RLK
LecRLK: Lectin-like RLK
LRR-RLK: Leucine-Rich Repeat RLK
PCD: Programmed Cell Death
PR5K-RLK: Pathogenesis related protein-5 like receptor kinases RLK
MEME: Multiple Em for Motif Elicitation
ML: Maxinum Likehood
RLK: Receptor-like protein Kinase
S-RLK: S-domain RLK
TNFR-RLK: Tumor-necrosis factor receptor-like RLK
WAK1: Cell wall-associated receptor kinase 1
