# Supplementary material for: Genome-wide identification and evolutionary analysis of RLKs involved in the response to aluminium stress in peanut
Source: BMC Plant Biol. 2021 Jun 21;21:281. doi: 10.1186/s12870-021-03031-4 (PMC8215822; doi:10.1186/s12870-021-03031-4)
Supplement: Supplementary file 8 — Additional file 8: The motif of Al stress-related AhRLKs. [file 12870_2021_3031_MOESM8_ESM.docx]

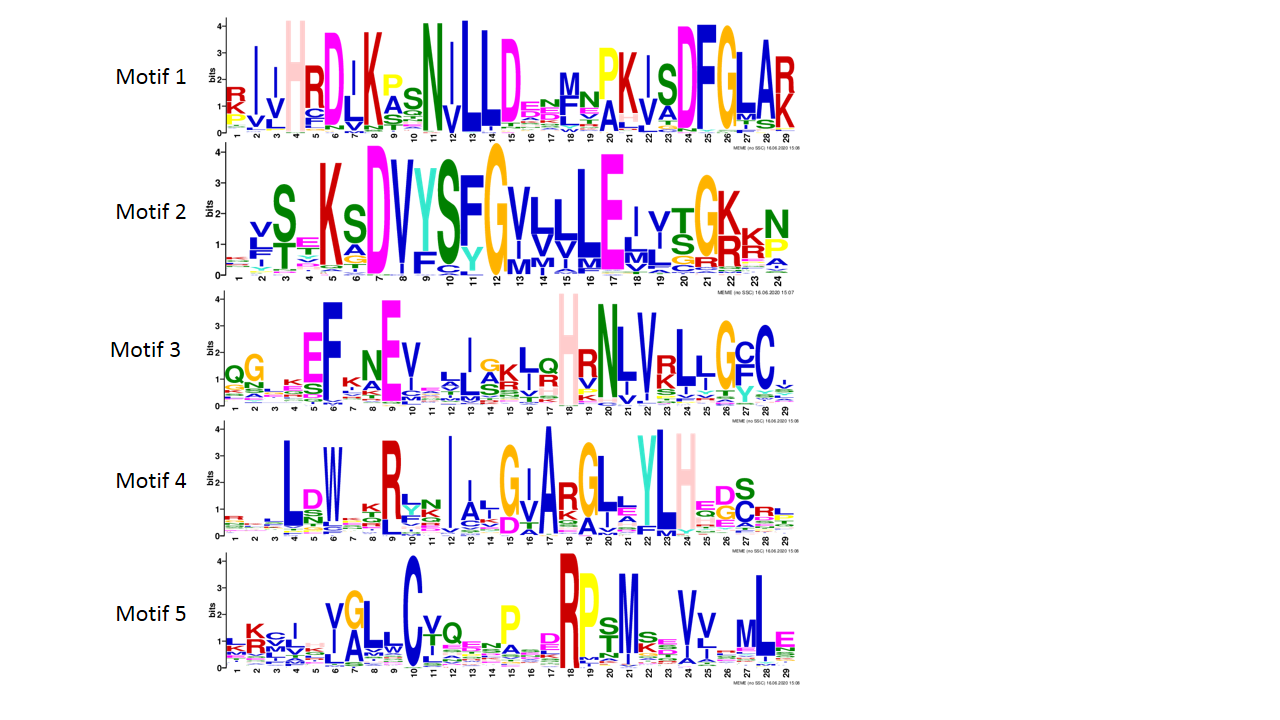


**Additional file 7: The motif of 90 *AhRLKs* in response to Al stress.**

Each column in the x-axis is composed of stack of letters where the height of these letters is indicative of the frequency of the letter at that position. The height of the stack is indicative of the sequence conservation.
